# Mesenchymal stem cell-derived exosomes enriched with miR-218 reduce the epithelial–mesenchymal transition and angiogenesis in triple-negative breast cancer cells

**DOI:** 10.1186/s40001-023-01463-2

**Published:** 2023-11-15

**Authors:** Samaneh Shojaei, Maryam Moradi-Chaleshtori, Mahdi Paryan, Ameneh Koochaki, Kazem Sharifi, Samira Mohammadi-Yeganeh

**Affiliations:** 1 Cellular and Molecular Biology Research Center, Shahid Beheshti University of Medical Sciences, Tehran, Iran; 2 Department of Medical Biotechnology, School of Advanced Technologies in Medicine, Shahid Beheshti University of Medical Sciences, Tehran, Iran; 3 Department of Research and Development, Production and Research Complex, Pasteur Institute of Iran, Tehran, Iran; 4 Medical Nanotechnology and Tissue Engineering Research Center, Shahid Beheshti University of Medical Sciences, Tehran, Iran

**Keywords:** Triple-negative breast cancer, Mesenchymal stem cells, Exosomes, miR-218, EMT, Angiogenesis

## Abstract

**Background:**

The epithelial–mesenchymal transition (EMT) and angiogenesis are morphogenetic processes implicated in tumor invasion and metastasis. It is found that the aberrant expression of microRNAs (miRNAs) contributes to these processes. Exosomes are considered potential natural vehicles for miRNA delivery in cancer therapy. miR-218 is one of the tumor suppressor miRNAs and its downregulation is associated with EMT and angiogenesis. We aimed to use adipose mesenchymal stem cells-derived exosomes (ADMSC-exosomes) for miR-218 delivery to breast cancer cells and evaluate miR-218 tumor-suppressing properties in vitro*.*

**Methods:**

Exosomes were isolated from conditioned media of ADMSCs. miR-218 was loaded to exosomes using electroporation. mRNA expression of target genes (*Runx2* and *Rictor*) in MDA-MB-231 breast cancer cells was evaluated by qPCR. To explore the effects of miR-218 containing exosomes on breast cancer cells, viability, apoptosis, and Boyden chamber assays were performed. The angiogenic capacity of MDA-MB-231 cells after treatment with miR-218 containing exosomes was assessed by in vitro tube formation assay.

**Results:**

miR-218 mimic was efficiently loaded to ADMSC-exosomes and delivered to MDA-MB-231 cells. Exposure to miR-218 containing exosomes significantly decreased miR-218 target genes (*Runx2* and *Rictor*) in MDA-MB-231 cells. They increased the expression of epithelial marker (*CDH1*) and reduced mesenchymal marker (*CDH2*). miR-218 restoration using miR-218 containing exosomes reduced viability, motility, invasion, and angiogenic capacity of breast cancer cells.

**Conclusions:**

These findings suggest that ADMSC-exosomes can efficiently restore miR-218 levels in breast cancer cells and miR-218 can prevent breast cancer progression with simultaneous targeting of angiogenesis and EMT.

## Introduction

Despite promising advancements in breast cancer therapeutic approaches, metastasis makes it the leading cause of cancer-related mortality [1], so the establishment of effective and safe therapeutic strategies is needed. Epithelial–mesenchymal transition (EMT) and angiogenesis are two critical processes for cancer cell metastasis.

MicroRNA (miRNA) profiling and deep sequencing indicate that aberrant expression of miRNAs in various cancer types is associated with cancer metastasis [2]. Restoring normal miRNA expression in cancer cells can change cancer phenotype. [3]. However, the administration of efficient and safe delivery systems is one of the main challenges in miRNA-based therapy [4].

Exosomes are membrane-bound nanovesicles produced by almost all cell types [5]. Unlike synthetic nanocarriers, exosomes do not face toxicity and immunogenicity as major drawbacks [6]. Low immunogenicity, high biosafety, and natural tumor tropism of mesenchymal stem cells (MSCs)-derived exosomes characterize them as an appropriate candidate for delivery of anti-cancer agents [7].

miR-218 is one of the tumor-suppressing miRNAs in different types of cancers [8–12]. There are conflicting reports on the role of miR-218 in breast cancer progression. Despite the studies showing the tumor-suppressing role of miR-218 in breast cancer [13–16], some studies suggest the tumor-promoting role of miR-218 [17, 18]. Therefore, the evaluation effects of miR-218, as a miRNA targeting EMT and angiogenesis, on breast cancer cells needs further studies.

In the present study, ADMSC-exosomes were used to overexpress miR-218 in breast cancer cells. We focused on RUNX family transcription factor 2 (*Runx2*) and RPTOR-independent companion of MTOR complex 2 (*Rictor*), two potential targets of miR-218 that play important role in EMT and angiogenesis.

## Methods

### ADMSCs isolation

To achieve enough number of cells from the best sources, adipose tissues were separately obtained from healthy donors (aged between 22 and 35 years) undergoing surgical procedures. They all signed an informed consent form approved by the ethics committee of Shahid Beheshti University of Medical Sciences (Ethical code: IR. SBMU.REC.1400.010).

Briefly, after washing adipose tissues from lipoaspirate samples with phosphate-buffered saline (PBS), they were digested with 0.1% collagenase I (Sigma, USA) for 40 min (min) at 37 °C with gentle agitation. At the end of the incubation time, collagenase was neutralized by adding FBS-containing medium and digested samples were centrifuged at 1200 RPM for 20 min (Hettich, Germany). The resultant cell pellet was resuspended in Dulbecco's Modified Eagle Medium/Nutrient Mixture F-12 (DMEM/F-12) supplemented with 15% FBS and 1% penicillin and streptomycin, seeded in culture flasks and maintained in a humidified atmosphere at 37 °C and 5% CO_2_. After 24 h, cells were washed to discard non-adherent cells and the fresh medium was replaced. During the expansion, half medium refreshment was done twice per week for optimal growth.

### ADMSCs characterization

Immunophenotyping of ADMSCs was performed using flow cytometry. At the third passage, 1 × 10^6^ ADMSCs were suspended in PBS and then incubated with primary antibodies including CD45-FITC, CD14-FITC, CD34-PE, CD90-FITC, CD73-PerCP, and CD105-PerCP (eBioscience, USA) for 30 min. Identification of ADMSCs’ surface markers was performed by FACSCalibur flow cytometer (BD Biosciences, USA).

The osteogenic and adipogenic differentiation potential were assessed using respective induction media and protocols [19]. ADMSCs (1 × 10^4^ cells/well) at passage 3 were seeded into 24-well plates and cultured in DMEM/F12 with 10% FBS. After 24 h, the differentiation media were replaced and refreshed every 3 days. 21 days after osteogenic induction, cells were fixed with 10% neutral formaldehyde and stained with 0.1% Alizarin red S dye (Sigma-Aldrich, USA). For adipogenic differentiation, after 14 days, cells were fixed and stained with 0.5% Oil Red O dye (Sigma-Aldrich, USA). The differentiated cells were observed by the light inverted microscope (Olympus, USA).

### Cell lines and culture conditions

HUVEC (human umbilical vein endothelial cells), MDA-MB-231 cells (triple-negative breast cancer cell line), and MCF-10A (non-tumorigenic breast cell line) were obtained from the Pasteur Institute of Iran (Tehran, Iran). MCF-10A and MDA-MB-231 cells were grown in DMEM containing 10% horse serum and FBS, respectively. HUVECs were cultured in DMEM/F-12 supplemented with 10% FBS. All cells were maintained in a humidified atmosphere at 37 °C and 5% CO_2_.

### Preparation of ADMSC-conditioned media and exosome isolation

The ADMSCs at the 3^rd^ passage were used for the collection of conditioned medium (CM). When cells reached 70–75% confluence, they were adapted to FBS-free medium containing 1% insulin–transferrin–selenium (ITS; Sigma, USA). Serum-free ADMSC-CM was collected after 72 h and used for exosomes isolation and characterization. AnnexinV/PI staining was performed to evaluate cell viability after 72 h serum starvation.

ADMSC-exosomes were isolated from ADMSC-CM using EXOCIB exosome purification kit (Cibbiotech, Iran) according to the manufacturer’s instructions. Briefly, the serum-free-CM was centrifuged at 3000 RPM for 10 min at room temperature to remove debris. Exosome precipitation solution was added to ADMSC-CM at a 1:5 (*v/v*) ratio and mixed by vortexing the tubes for 5 min and incubated overnight at 4 °C. Then, the samples were centrifuged at 3000 RPM for 40 min at 4 °C. After removing the supernatant, exosomes were resuspended with PBS for the following experiments.

### Exosome characterization

The size distribution of extracted exosomes was determined using dynamic light scattering (DLS) Zetasizer (Malvern, UK). The morphology and size of exosomes were observed using transmission electron microscopy (TEM) and scanning electron microscopy (SEM). For TEM imaging (Zeiss EM900), after processing of ADMSC-exosomes (fixation, dehydration, and sectioning), the ultrathin sections were prepared and stained using uranyl acetate and lead citrate and visualized under electron microscopy. For SEM (KYKY-EM3200, China), after fixation and dehydration of exosomes, they were left on the glass substrate to dry at room temperature and then were analyzed by scanning electron microscope.

### Loading ADMSC-exosomes with miRNA mimic

To load miRNA-218-5p mimics (Bioneer, Korea) into ADMSC-exosomes, electroporation method was used. ADMSC-exosomes at a final concentration of 100 µg/µl protein (measured by BCA) were mixed with electroporation buffer (in a 1:1 ratio) and 100 pmol of synthetic miR-218 mimics or negative control (Scramble), and electroporated at 0.400 kV using an electroporation instrument (Eppendorf, Germany). To evaluate the efficiency of the loading protocol, MDA-MB-231 cells were incubated with 100 µg/ml of manipulated exosomes for 48 h and miR-218 encapsulation in exosomes was quantified using qRT-PCR.

The experimental groups included MDA-MB-231 cells treated with miR-218 or scramble containing exosomes, cells treated with unmodified exosomes, and untreated cells.

### RNA extraction, cDNA synthesis, and qPCR

Total RNA from MDA-MB-231 cells treated with modified exosomes and their controls was extracted after 48 h using Hybrid-R^™^ (GeneAll, Korea) according to the manufacturer’s instructions. Using RT-Stem loop [20] and random hexamer primers, the RNA of miR-218 and related genes [*Runx2*, *Rictor*, vascular endothelial growth factor A (*VEGF*), cadherin 1 or E-cadherin (*CDH1*), cadherin 2 or N-cadherin (*CDH2*)] were, respectively, transcribed to complementary DNA (cDNA).

Relative expression of miR-218 and target mRNAs was evaluated using TaqMan® probe and SYBR Green I Master Mix (Amplicon, Germany), respectively, in a StepOne instrument (Applied Biosystems, USA). SNORD47 and β-actin were considered as the internal references for miR-218 and mRNAs, respectively. The 2 ^–ΔΔCt^ method was employed to compute the relative expression of miRNA and mRNAs.

### MTT assay

MDA-MB-231 cells were plated in a 96-well plate (3 × 10^3^ cells/well) and incubated in serum-containing DMEM. After overnight incubation, cells were treated with modified exosomes (100 µg/ml) and their controls in a serum-free medium. At defined time points, the medium was removed and cells were incubated with 100 µL of MTT solution in PBS (0.5 mg/mL) for 3 h in the cell culture incubator. After removing the supernatant, 100 µL of dimethyl sulfoxide (DMSO) was added to each well for 2 h to dissolve formazan crystals. The optical density was read at 570 nm using a microplate reader (BioTek, USA).

### Annexin V/PI assay

MDA-MB-231 cells were plated in a 24-well plate (5 × 10^4^ cells/well). 48 and 72 h after their incubation with exosomes (100 µg/ml) in respective groups, cells were trypsinized and stained with annexin V/ fluorescein isothiocyanate (FITC) / propidium iodide (PI) kit according to the manufacturer’s instructions (Abcam, US). FACSCalibur flow cytometer was employed for cell analysis (BD Biosciences, USA). The annexin V^+^/PI^−^ and annexin V^+^/PI ^+^ cells were considered as early and late apoptotic cells, respectively.

### Scratch assay

MDA-MB-231 cells (12 × 10^4^ cells/well) were plated in a 24-well plate. On reaching 90–95% confluence, the scratch was made across each well using a sterile 100 µl tip. After washing with DMEM to remove cell debris, cells were treated with 100 µg/ml modified exosomes in serum-free medium. Plates were photographed by an inverted microscope for 48 h and the images were processed and quantified using the ImageJ software (NIH, USA).

### Cell migration assay

5 × 10^4^ MDA-MB-231 cells suspended in serum-free medium with modified exosomes (100 µg/ml) were added into the upper chamber of transwell inserts (24-well insert; pore size 8 µm; SPL) and exposed to FBS-containing medium (as a chemoattractant) in the bottom chamber for 48 h. After incubation time, non-migrated cells were removed by scraping the upper surface of the chamber, and inserts were fixed and then stained with crystal violet. Five random fields were selected for counting the number of migrated cells.

### Cell invasion assay

5 × 10^4^ MDA-MB-231 cells were seeded to Matrigel (Corning, USA) coated inserts. Matrigel, being prepared by mixing with serum-free DMEM at a ratio of 1:2, was added to inserts and maintained at 37 °C for 2 h to solidify. The next steps were similar to those described for the migration assay.

### In vitro* angiogenesis assays*

HUVECs were plated in a 96-well plate (3 × 10^3^ cells /well) and incubated in DMEM-F12 overnight. Then, the culture medium was replaced with conditioned media of breast cancer cells treated with modified ADMSC-exosomes and their controls. After 24, 48, and 72 h, viable cells were evaluated by MTT assay.

In vitro migration assay was performed as described above. 4 × 10^4^ HUVECs were added in the upper chamber of transwell inserts and cultured in serum-free DMEM-F12. The conditioned media of breast cancer cells treated with exosomes-encapsulated miR-218 and their controls were added to the lower chamber. After 24 h, migrated cells were stained and counted in five randomly selected microscopic fields.

In vitro capillary network formation was evaluated by tube formation assay. 3 × 10^4^ cells were seeded into Matrigel-coated 48-well plate. Next, the cells were incubated with conditioned media collected from breast cancer cells treated with miR-218 containing exosomes and their controls for 24 h. The number of meshes and total branching length were quantified by randomly selecting five fields per well by using angiogenesis analyzer ImageJ plugin (NIH, USA).

### Statistical analysis

Statistical analysis was conducted using GraphPad Prism (GraphPad, San Diego, CA). Student’s *t-test* was used for comparison between two groups while data among multiple groups were compared by one-way ANOVA. All experiments were performed in triplicate. The data were finally presented as mean ± SD and the asterisks show significant p-value: * *p* < 0.05; ** *p* < 0.001; *** *p* < 0.0001; and **** *p.* < 0.00001).

## Results

### ADMSC characterization

**Fig. 1:**
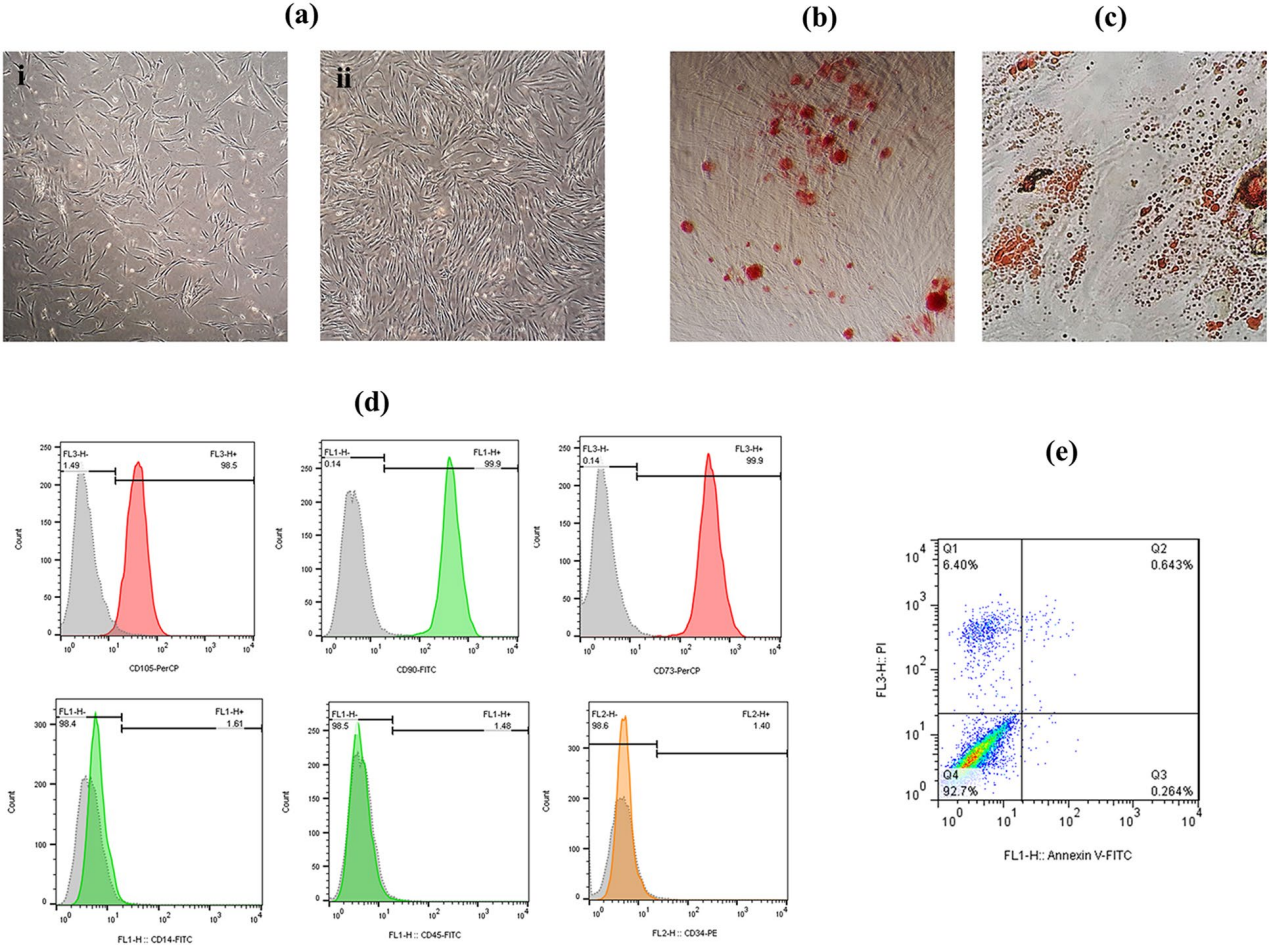
Characterization of ADMSC. **a** Microscopic images of the first (i) and the fourth (ii) generations of ADMSCs. **b** Alizarin red staining after 3 weeks of osteogenic induction in ADMSCs. **c** Oil red O staining after 2 weeks of adipogenic differentiation in ADMSCs. **d** Positive markers (CD105, CD90, CD73) and negative markers (CD45, CD34, CD14) of ADMSCs identified by flow cytometry. **e** The viability of the ADMSCs 72 h after serum starvation

ADMSCs appeared, morphologically, as a homogeneous population of spindle-shaped cells during the initial passages (Fig. 1a). The differentiation capacity of ADMSCs toward osteogenic and adipogenic fates was confirmed using respective conditioned media (Fig. 1b, c). Immunophenotyping of ADMSCs using flow cytometry confirmed that ADMSCs were positive for mesenchymal markers (CD 73, CD 90, and CD 105) and negative for haematopoietic markers (CD 14, CD 34, and CD 45) (Fig. 1d). Besides, annexin V/PI assay revealed that 92.7% of ADMSCs were viable after 72 h of serum starvation (Fig. 1e).

### Exosome characterization

**Fig. 2:**
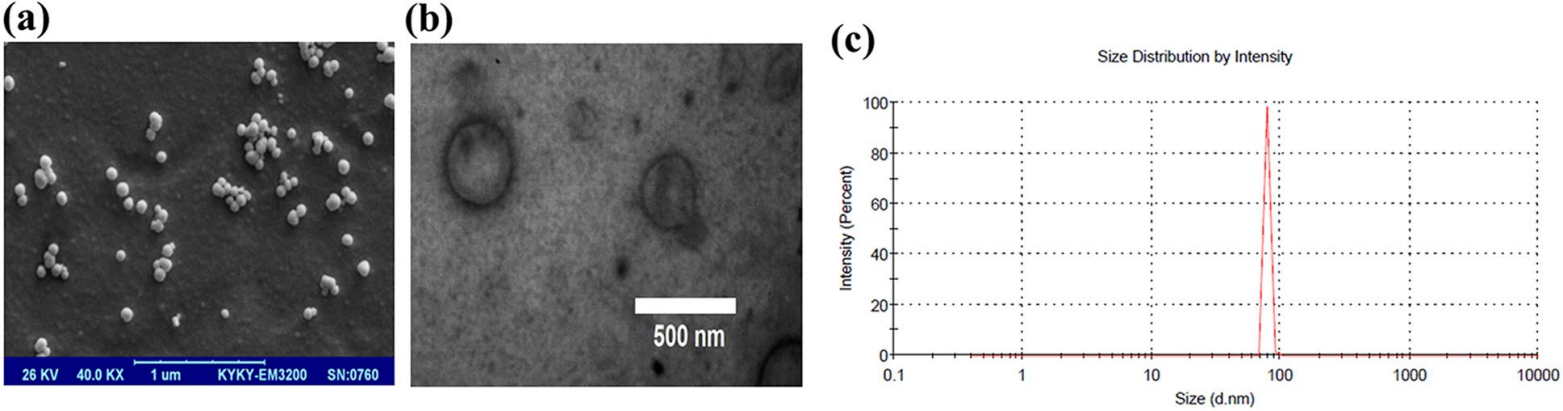
Characterization of ADMSC-exosomes. **a** The ultrastructure observed by scanning electron microscopy (SEM). **b** Transmission electron microscopy (TEM) micrograph of ADMSC-exosomes. **c** Size distribution of exosomes detected by dynamic light scattering

We verified morphology, size, and specific markers of isolated exosomes using different methods. SEM and TEM imaging identified vesicles with a diameter of 40–100 nm (Fig. 2a) and complete membrane structures (Fig. 2b). As shown in Fig. 2c, the size distribution analysis of ADMSC-exosomes revealed that the average size of exosomes was 90 nm.

### miR-218 is poorly expressed and Runx2 and Rictor are highly expressed in MDA-MB-231 cells

**Fig. 3:**
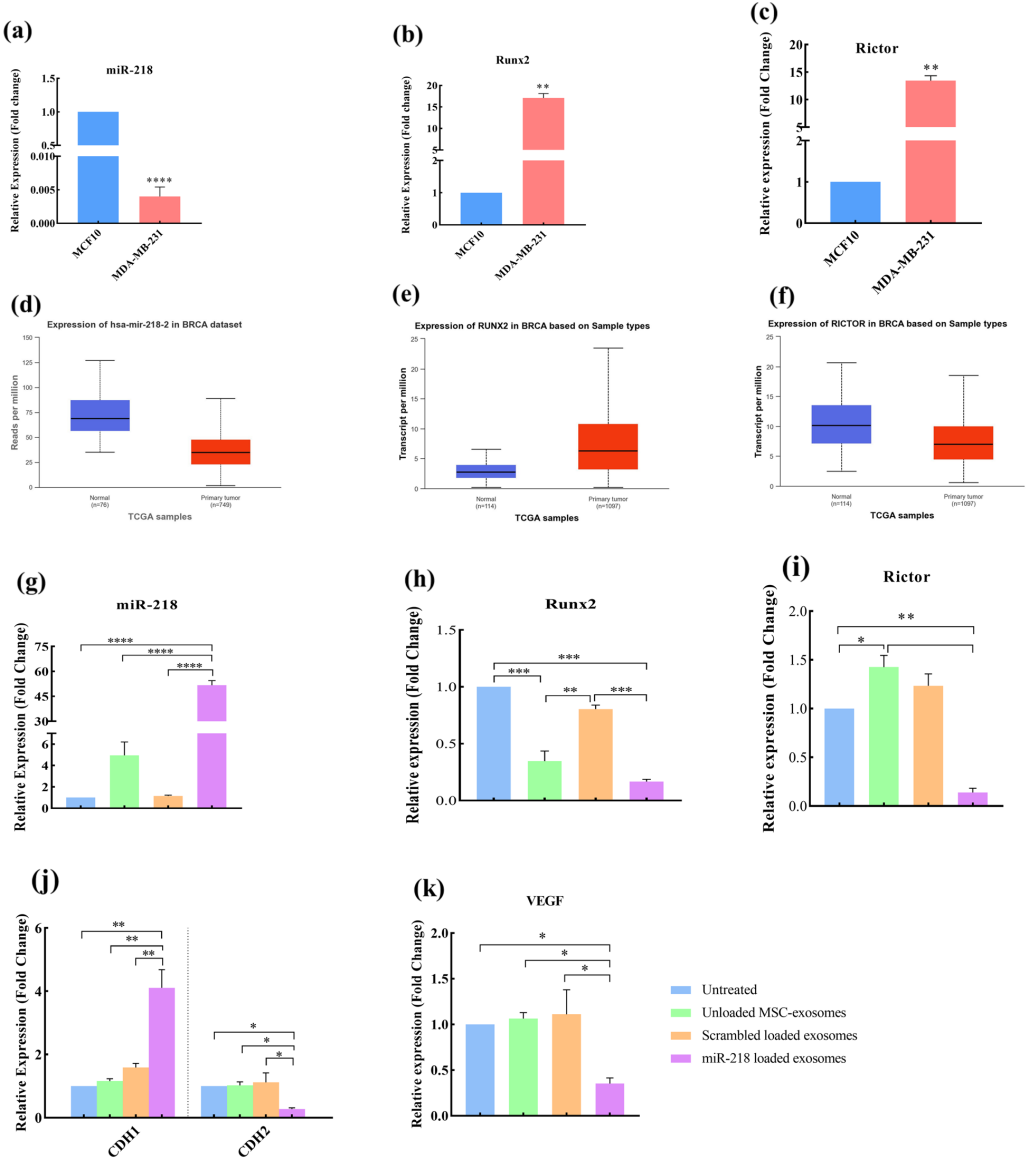
Gene expression analysis. **a** The relative expression of miR-218 level in MDA-MB-231 cells compared to normal MCF10A cells. **b** The relative expression of *Runx2* in MDA-MB-231 cells compared to normal MCF10A cells**. c** The relative expression of *Rictor* in MDA-MB-231 cells compared to normal MCF10A cells. The expression of miR-218 **(d)**, *Runx2* (**e**) *Rictor* **(f)** in The Cancer Genome Atlas (TCGA) of breast cancer samples in UALCAN database. **g** The relative expression of miR-218 level in MDA-MB-231 cells treated with miR-218 containing ADMSC-exosomes and their controls determined by qRT-PCR. The relative expression of *Runx2* **(h)**, *Rictor* **(i)**, E-cadherin *(CDH1)* and N-cadherin *(CDH2)* **(j)**, and VEGF(**k**) mRNA levels in MDA-MB-231 cells treated with miR-218 containing ADMSC-exosomes and their controls determined by qRT-PCR. The data are presented as mean ± SD (*p value < 0.05, **p value < 0.001, and ***p < 0.0001, ****p < 0.00001)

Because of the conflicting results reported in miR-218 expression in breast cancer cells in previous studies, we evaluated the expression of miR-218 in invasive breast cancer cell line, MDA-MB-231, compared to normal human breast cancer cell line, MCF-10A, and results demonstrated that miR-218 was decreased significantly in MDA-MB-231 (Fig. 3a). Among the validated target genes of miR-218 in online miRNA databases, we focused on *Runx2* and *Rictor* because of their significant role in the EMT and angiogenesis. We assessed *Runx2* and *Rictor* mRNA expression in MDA-MB-231 cells compared to MCF-10A cells by qPCR and found that the expression of *Runx2* (Fig. 3b) and *Rictor* (Fig. 3c) was markedly higher in MDA-MB-231 cells.

Additionally, we have examined the expression of miR-218, *Runx2*, and *Rictor* in breast cancer in The Cancer Genome Atlas (TCGA) samples in UALCAN database. As expected, the expression of miR-218 is decreased (Fig. 3d) and the expression of *Runx2* is increased (Fig. 3e) in breast cancer samples compared to normal samples. In the case of *Rictor*, the results were contrary to expectation. According to TCGA data, the expression of *Rictor* in breast cancer samples has decreased compared to normal samples (Fig. 3f).

### miR-218 containing exosomes downregulate the expression of key mediators of EMT and angiogenesis

ADMSC-exosomes were used to transfer miR-218 mimic to breast cancer cells. The efficiency of miR-218 delivery to breast cancer cells via exosomes and its effects on target genes was examined by qRT-PCR. The results demonstrated that miR-218 containing exosomes considerably increased miR-218 levels in MDA-MB-231 cells (Fig. 3g). Furthermore, miR-218 delivery via ADMSC-exosomes led to a significant decrease in *Runx2* (Fig. 3h) and *Rictor* (Fig. 3i) mRNAs levels in breast cancer cells. Since Runx2 act as an EMT transcription factor [21, 22], we evaluated the expression of EMT-related markers in MDA-MB-231 cells and observed that the expression of mesenchymal marker, *CDH2*, significantly decreased while the expression of epithelial marker, *CDH1*, remarkably increased after incubation with miR-218 containing exosomes (Fig. 3j). As Runx2 and Rictor play key roles in angiogenesis, the expression of *VEGF* was also assessed in breast cancer cells after treatment with miR-218 containing exosomes. The results indicated low *VEGF* levels in cells treated with miR-218 containing exosomes compared to controls (Fig. 3k). These results show that exosomal delivery of miR-218 reduces the expression of major mediators of EMT and angiogenesis.

### miR-218 delivery using ADMSC-exosomes reduced viability of breast cancer cells

**Fig. 4:**
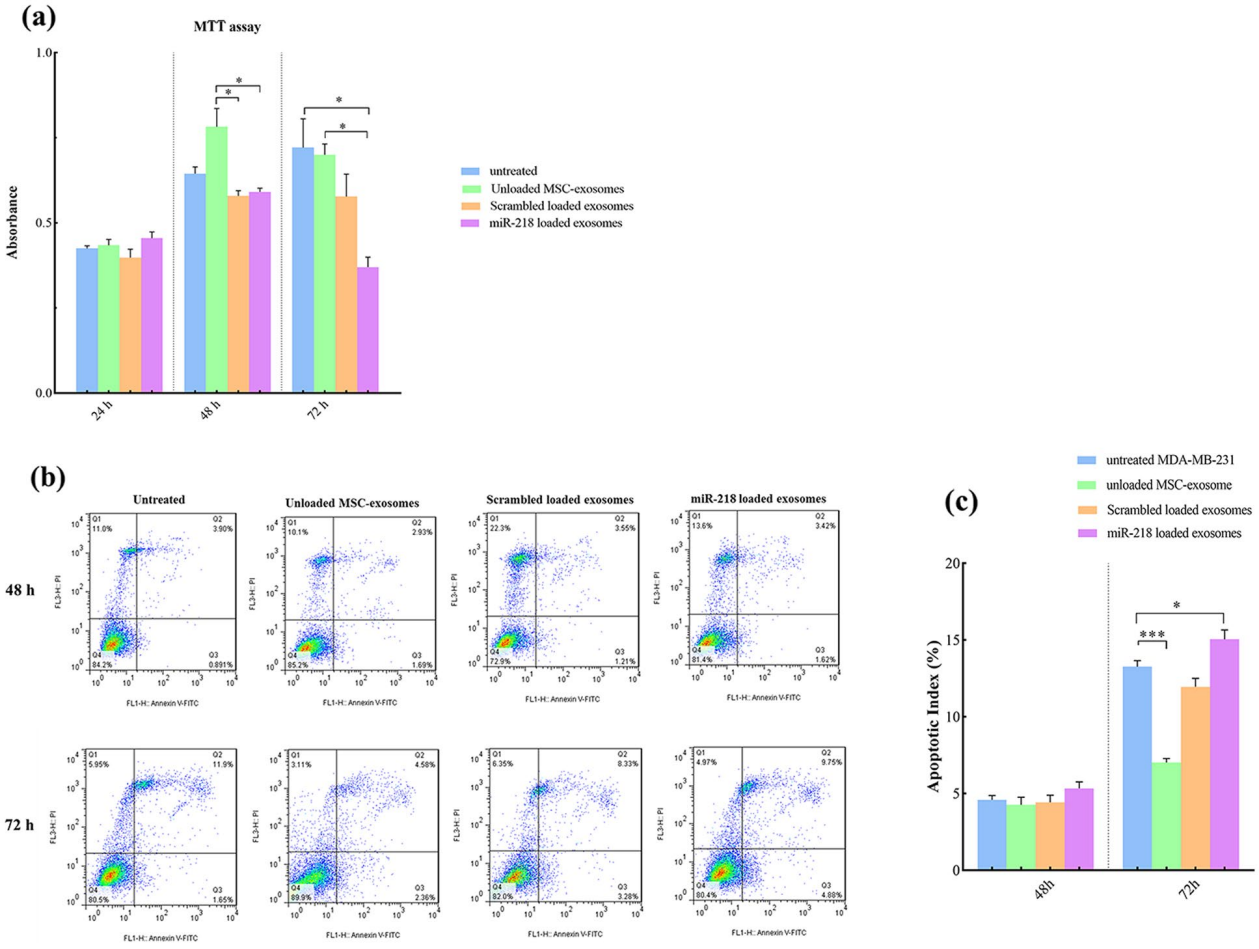
miR-218 containing exosomes reduce viability of MDA-MB-231 cells in vitro*.* **a** Bar graphs showing the MTT assay for analyzing the effect of miR-218 containing exosomes on the viability of MDA-MB-231 cells. **b** Representative dot plots showing the percentage of apoptotic cells in MDA-MB-231 cells 48 h and 72 h after treatment with miR-218 containing exosomes and their controls, detected by Annexin V/PI flow cytometry assay. **c** Bar graphs showing the apoptotic index detected by Annexin V/PI flow cytometry assay represented in (b), shown as mean ± SD from 3 independent experiments. (*p < 0.05, and *** *p* < 0.0001)

The effect of miR-218 containing exosomes on breast cancer cell viability was determined by MTT assay. miR-218 containing exosomes (after 24 and 48 h) had no significant effect on the total number of viable cells compared to untreated cells. However, a significant decrease on the total number of viable cells was observed upon treatment with miR-218 containing exosomes after 72 compared with controls (Fig. 4a). A notable finding was a non-significant increase in the total number of viable cells 48 h after treatment with unmodified exosomes.

To further confirm the effects of treatment with miR-218 containing exosomes on the viability of breast cancer cells, Annexin V/PI assay was performed as a specific viability test. Although after 48 h, the apoptosis rates were not significantly different among experiment groups, the apoptosis rate significantly increased in breast cancer cells after 72 h exposure to miR-218 containing exosomes. Surprisingly, the apoptosis percentage of breast cancer cells significantly decreased after 72 h incubation with unmodified ADMSC-exosomes (Fig. 4b and c).

### *Migration and invasion of breast cancer cells are inhibited after delivery of miR-218 *via* ADMSC-exosomes*

**Fig. 5:**
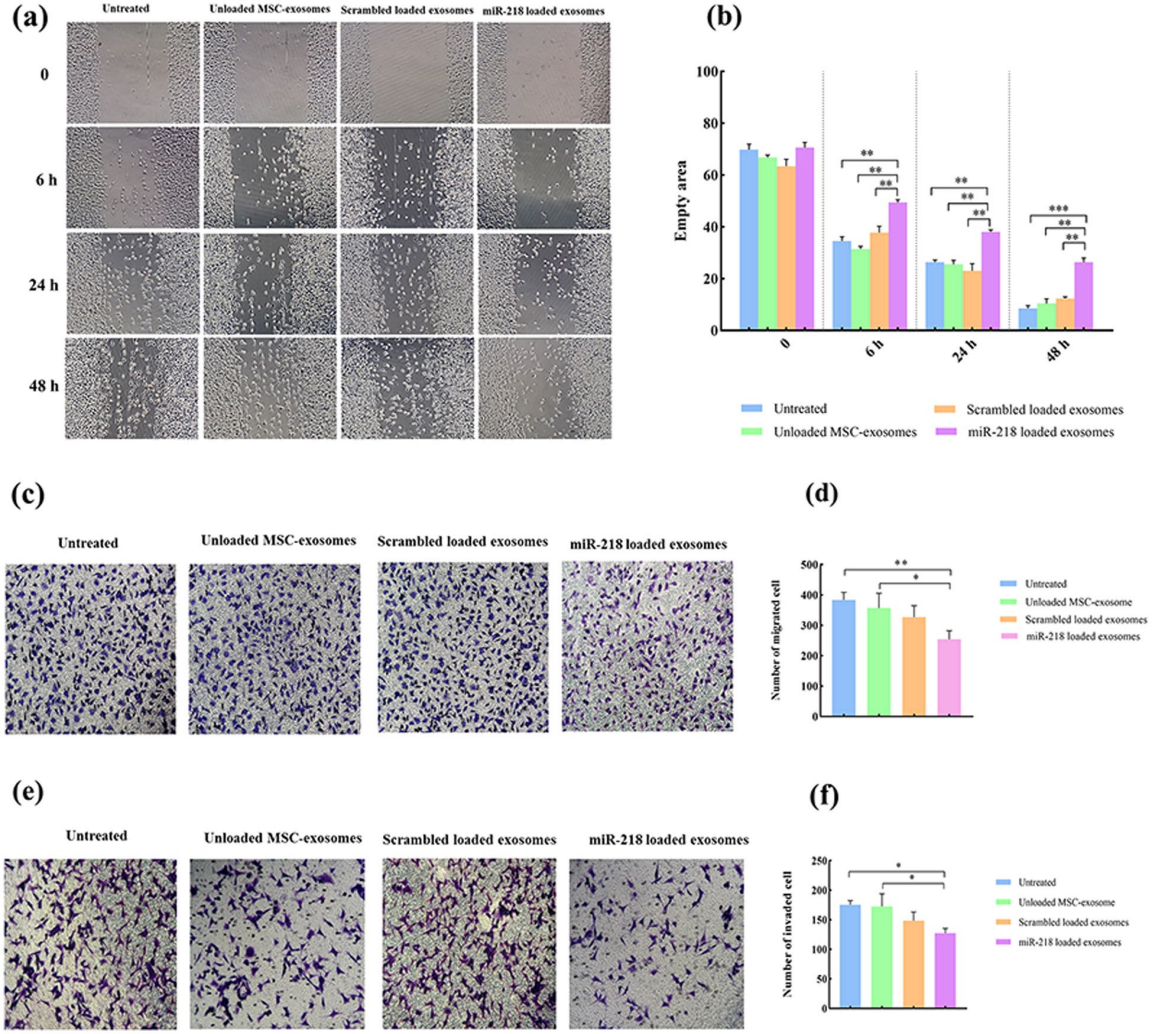
miR-218 containing exosomes inhibit migration and invasion of MDA-MB-231 cells in vitro. **a** Representative photomicrographs showing the scratch assay of MDA‐MB‐231 cells treated with miR‐218 containing exosomes. **b** Bar graphs showing the significant decrease in motility of the cells treated with miR‐218 containing exosomes compared with control. **c** Representative photomicrographs showing the transwell migration assay of MDA‐MB‐231 cells treated with miR‐218 loaded exosomes. **d** Bar graphs showing the significant decrease in migration of the cells treated with miR‐218 containing exosomes. **e** Representative photomicrographs showing the transwell invasion assay of MDA‐MB‐231 cells treated with miR‐218 containing exosomes. **f** Bar graphs showing the significant decrease in invasion of the cells treated with miR‐218 loaded exosomes. Data are shown as mean ± SD from 3 independent experiments. (*p < 0.05 and **p < 0.001)

We investigated the effects of miR-218 containing exosomes on migratory and invasive properties of breast cancer cells via performing the scratch and transwell migration/invasion assays. The results of the scratch assay demonstrated that cells treated with miR-218 containing exosomes did not fill the scratch after 48 h (Fig. 5a and 5b). We conducted transwell assays to further confirm the effects of miR-218 containing exosomes on breast cancer cell motility. Similar results were observed in the transwell migration assay and treatment with miR-218 containing exosomes led to a considerable reduction in migration of breast cancer cells compared to controls (Fig. 5c-d). Also, the number of invaded cells in the miR-218 treated cells showed a significant decrease compared to untreated or unmodified exosome-treated cells. Our findings indicated that miR-218 delivery via ADMSC-exosomes significantly reduced the motility and invasiveness of breast cancer compared to the control groups.

### *miR-218 containing ADMSC-exosomes decrease the angiogenic capacity of breast cancer cells *in vitro

**Fig. 6:**
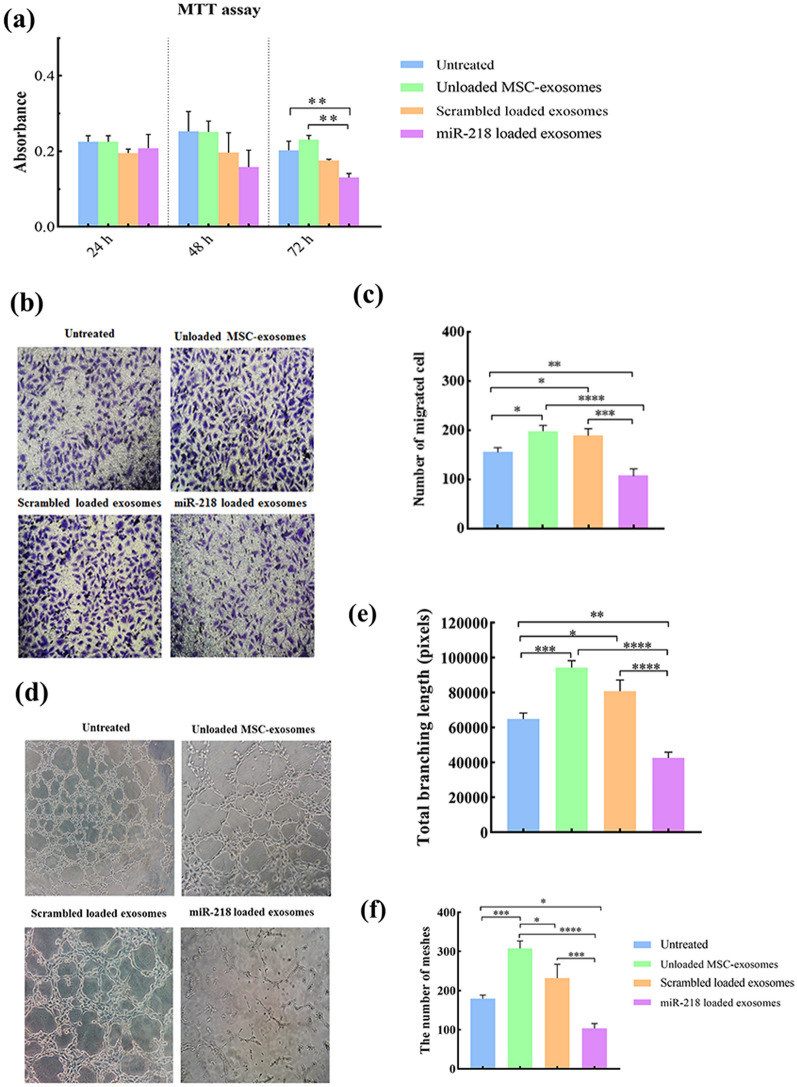
miR-218 containing exosomes reduce angiogenic capacity of MDA-MB-231 cells. **a** Bar graphs showing the MTT assay for analyzing the effect of miR-218 containing exosomes on the viability and proliferation of HUVECs. **b** Representative photomicrographs showing the transwell migration assay of HUVECs after incubation with CM of exosome-treated MDA-MB-231 cells. **c** Bar graphs showing the significant decrease in migration of the cells incubated with CM of miR‐218 treated MDA-MB-231 cells. **d** Representative photomicrographs showing the tube formation of HUVECs treated with CM of exosome-treated MDA-MB-231 cells. **e–f** Bar graphs showing the number of meshes and total branching length were analyzed about the blood vessel formation that showed significant decrease in HUVECs treated with CM of exosome-treated MDA-MB-231 cells. Data are shown as mean ± SD from 3 independent experiments. (*p < 0.05, **p < 0.001, and ***p < 0.0001, ****p< 0.00001)

To test whether treatment of breast cancer cells with miR-218 containing exosomes can affect their angiogenic potential, we incubated endothelial cells with conditioned media of exosome-treated MDA-MB-231 cells and evaluated their viability, migration, and tube formation in vitro. As shown in Fig. 6a, 24 and 48 h after incubation of endothelial cells with CM of breast cancer cells, no significant difference was observed in cell viability. However, after 72 h, a significant decrease was observed in the number of viable HUVECs incubated with CM of miR-218-treated breast cancer cells. The migration capacity of HUVECs was decreased upon incubation with CM of MDA-MB-231 cells treated with miR-218 containing exosomes compared with control CMs (Fig. 6b-c). We also analyzed the newly formed vascular structures quantitatively. The number of meshes and total branching length were significantly lower in HUVECs incubated with CM of miR-218-treated MDA-MB-231 cells compared to control groups (Fig. 6d-f). Interestingly, the CM of MDA-MB-231 cells incubated with unloaded ADMSC-exosomes significantly increased the migration and tube formation of endothelial cells.

## Discussion

Delivery of miRNAs inhibiting EMT and angiogenesis via clinically applicable vehicles can be a promising therapeutic strategy in metastatic cancers. Recently, numerous studies including our previous reports have focused on the therapeutic potential of MSC-derived exosomes for appropriate delivery of oligonucleotides in cancer therapy [19, 23, 24]. In the present study, we selected miR-218 as a potential therapeutic candidate and used ADMSC-exosomes for its effective delivery to breast cancer cells. We showed that miR-218 overexpression using ADMSC-exosomes impaired breast cancer cells migration, invasion, and viability and downregulated mediators and markers of EMT and angiogenesis.

miR-218 acts as a tumor suppressor miRNA in various types of cancer and its downregulation is associated with tumor progression and metastasis [9–11, 25]. We demonstrated that miR-218 is downregulated in invasive MDA-MB-231 cells compared to normal MCF-10 cells. This result is in accordance with the previous findings showing that miR-218 is downregulated in breast cancer cells [14] and in contrast to miR-218 overexpression in breast cancer cells shown by Liu et al. [17]*.*

*Runx2* and *Rictor* are two potential targets of miR-218. It was previously confirmed that miR-218 targets 3'UTR of *Runx2* and *Rictor* [10, 26] but the effects of their interaction have not yet been reported in breast cancer. In this study, the breast cancer cells expressed high levels of *Runx2* and *Rictor* compared to non-tumorigenic MCF-10A cells which were in line with previous studies [27, 28]. The expression of *Runx2* is disrupted in breast cancer and induces the expression of EMT-related transcription factors and metastasis-related genes [29, 30].

Runx2 directly or indirectly increases the expression of vascular endothelial growth factor (VEGF), the most important angiogenic factor [31, 32]. The indirect effect of Runx2 on *VEGF* expression is mediated by Rictor, the major component of mTORC2 [26]. *Rictor* is amplified and upregulated in breast cancer [33, 34]. Rictor is involved in multiple myeloma and prostate cancer angiogenesis and its inhibition suppresses tumor angiogenesis [26, 35].

In this study, we found that restoration of miR-218 using miR-218 containing exosomes downregulates *Runx2* in breast cancer cells at the mRNA levels. Our results were consistent with previous reports in thyroid and ovarian cancers in which miR-218 overexpression via lipid-based transfection downregulates *Runx2* and inhibits cell proliferation, migration, and invasion in vitro [10, 12]. Furthermore, we indicated that miR-218 overexpression using miR-218 containing exosomes downregulates *Rictor* and *VEGF* in breast cancer cells. A similar result has been shown by Guan et al*.* in prostate cancer. They showed that miR-218 overexpression by lentiviral vectors restrains tumor angiogenesis via targeting *Rictor*/*VEGF* axis [26].

Overexpression of miR-218 using miR-218 containing exosomes reduces motility and invasiveness of MDA-MB-231 cells in vitro as previously shown by Setijono *et al* [14]l. The anti-angiogenic role of miR-218 and the pro-angiogenic properties of Runx 2 and Rictor [31, 32] led us to examine the angiogenic effects of MDA-MB-231 cells treated with miR-218 containing exosomes on endothelial cells. Our data showed that conditioned media of breast cancer cells treated with miR-218 containing exosomes significantly decreased viability, migration, and tube formation of endothelial cells compared to other groups. This finding was consistent with our gene expression analysis in which the expression of *VEGF* mRNA in MDA-MB-231 cells treated with miR-218 containing exosomes decreased significantly.

A significant finding in this study was that unmanipulated ADMSC-exosomes reduced the apoptosis of breast cancer cells. This finding can be attributed to the presence of large amounts of negative regulators of the apoptosis process in unmanipulated ADMSC-exosomes [36]. The anti-apoptotic activity of ADMSC-secretome has also been shown in liver injury [37]. Moreover, as shown in previous reports, our results indicated that unmanipulated ADMSC-exosomes increase angiogenesis in vitro [38, 39].

Generally, exosomes derived from MSCs of different tissue origins have shown promising results in miRNA delivery and inhibiting breast cancer development. Exosomes derived from miRNAs-overexpressing bone marrow-MSC inhibit breast cancer cell invasiveness and angiogenesis [40–44]. Delivery of exogenous miRNA by exosomes derived from umbilical cord- MSC suppresses tumor invasion in breast cancer (23).

## Conclusions

We conclude that ADMSC-exosomes can efficiently deliver miRNA to tumor cells. We suggest that miR-218 loaded ADMSC-exosomes may be an effective anti-metastatic and anti-angiogenic treatment in breast cancer in part through targeting *Runx2* and *Rictor*. However, this study has its own limitations, including the lack of an animal model and confirmation of gene expression data in protein level. To increase the credibility of results, concentrating on more cell lines, more in vitro experiments such as colony formation assay, in vivo experiments, and detailed mechanisms of miRNA function in cancer signaling pathways are recommended.

## Data Availability

All data generated or analyzed during this study are included in this manuscript. Raw data will be made available on a reasonable request from the corresponding author.
